# Neuropsychiatric and Hematologic Manifestations in Nitrous Oxide Psychosis: A Case Report and Scoping Review

**DOI:** 10.1192/j.eurpsy.2025.968

**Published:** 2025-08-26

**Authors:** S. Liaqat, F. Rizwan Bhatti, A. Deka

**Affiliations:** 1 Psychiatry, RUSH University Medical Center, Chicago, United States; 2 Psychiatry, Pakistan Institute of Medical Sciences, Islamabad, Pakistan

## Abstract

**Introduction:**

Nitrous Oxide (N2O) has recently emerged as a global public health threat, particularly in young adults resulting in neuropsychiatric and hematologic sequelae.

**Objectives:**

This study aims to highlight a case of N2O-induced psychosis with a review of literature on neuropsychiatric and hematologic complications with N2O use in patients presenting with psychosis, with elucidation of mechanisms, clinical presentations and management.

**Methods:**

Case was reviewed, and findings were contextualized with existing literature. Relevant keywords yielded 253 studies; after title and abstract screening, 14 studies were reviewed.

**Results:**

A 33-year old male with no known comorbidities presented with bizarre behaviors with paranoia, persecutory delusions, delusion of control, ideas of reference, Cotard delusion, and hallucinations. He reported taking daily B12 supplements because he was aware of the effects of N2O on B12 metabolism. Collateral information revealed N2O use for >5 years with recent escalation. Labs revealed high homocysteine (93.45 μmol/L), despite normal B12 and MMA level. Syphilis was negative. Neurological examination showed intact proprioception, vibration and gait, and a negative Romberg sign. MRI indicated nonspecific FLAIR hyperintensities. He was admitted to the inpatient psychiatric unit where he initially remained disorganized, paranoid, and attempting to elope, requiring PRN medications. Risperidone was uptitrated to 0.5 mg QAM and 1 mg QHS, while continuing B12 supplementation. On discharge, psychosis had resolved. He was provided psychoeducation regarding the complications of N2O use, he demonstrated understanding, however, remained in pre-contemplation stage.

Literature review (Fig 1) highlights significant neuropsychiatric and hematological effects. B12 deficiency resulting in sensorimotor polyneuropathy and axonal degeneration, with MRI often showing symmetrical cervical spinal cord abnormalities has been reported in multiple studies. EMG tests reveal peripheral nerve damage. Hematologically, it is linked to cerebral venous sinus thrombosis and DVT, with elevated homocysteine raising thrombosis risk (Fig 2). Psychiatrically, nitrous oxide can cause transient psychosis and suicidal behavior, although cases of subsequent primary psychotic illness have been reported. Management includes cessation of use, B12 supplementation; occasional use of antipsychotics has also been reported.

**Image:**

**Figure:**
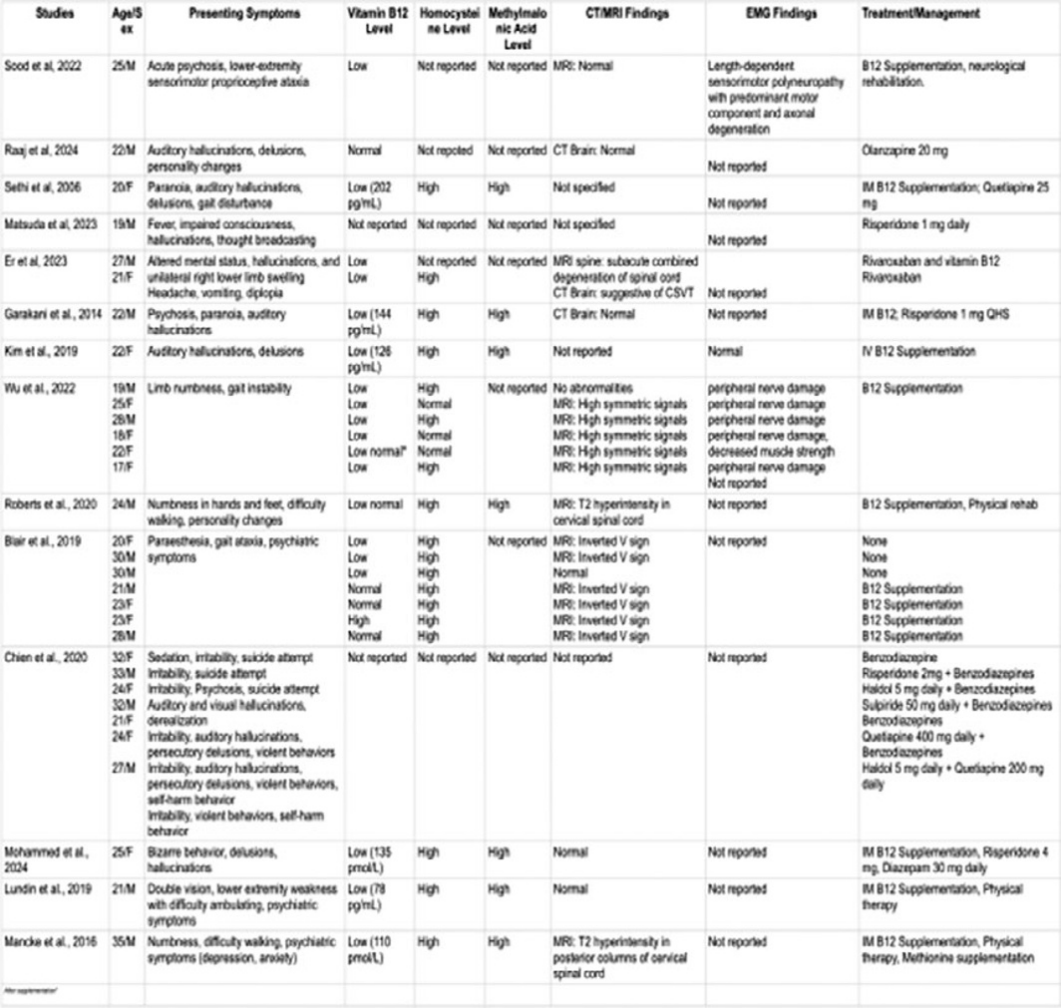

**Image 2:**

**Figure:**
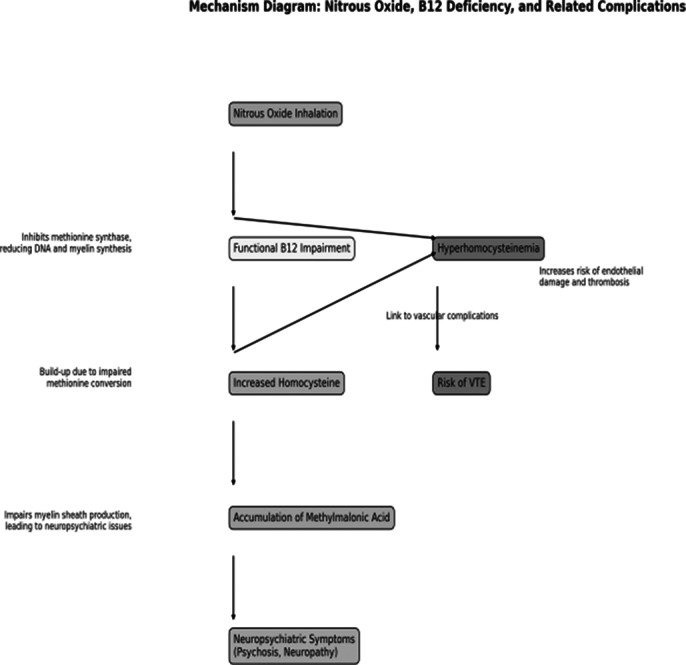

**Conclusions:**

The patient’s presentation underscores the complexity of N2O-induced neuropsychiatric and hematologic conditions. The findings emphasize the need for thorough clinical evaluations, including psychiatric and neurologic assessments, and laboratory testing when N2O use is suspected. Future research should focus on early recognition and intervention, optimizing management strategies, and understanding the long-term prognosis of patients with N2O-induced psychosis and associated sequelae.

**Disclosure of Interest:**

None Declared

